# The genome of a prasinoviruses-related freshwater virus reveals unusual diversity of phycodnaviruses

**DOI:** 10.1186/s12864-018-4432-4

**Published:** 2018-01-15

**Authors:** Hao Chen, Weijia Zhang, Xiefei Li, Yingjie Pan, Shuling Yan, Yongjie Wang

**Affiliations:** 1 College of Food Science and Technology, Shanghai Ocean University, Shanghai, China; 2 Laboratory for Marine Biology and Biotechnology, Qingdao National Laboratory for Marine Science and Technology, Qingdao, China; 3 Laboratory of Quality and Safety Risk Assessment for Aquatic Products on Storage and Preservation, Ministry of Agriculture, Shanghai, China; 4 Institute of Biochemistry and Molecular Cell Biology, University of Göttingen, Göttingen, Germany; 5 Present address: Archaea Center, Department of Biology, Copenhagen University, DK2000 Copenhagen, Denmark

**Keywords:** *Phycodnaviridae*, *Prasinovirus*, DSLPV1, Histone H3, Diversity

## Abstract

**Background:**

Phycodnaviruses are widespread algae-infecting large dsDNA viruses and presently contain six genera: *Chlorovirus*, *Prasinovirus*, *Prymnesiovirus*, *Phaeovirus*, *Coccolithovirus* and *Raphidovirus.* The members in *Prasinovirus* are identified as marine viruses due to their marine algal hosts, while prasinovirus freshwater relatives remain rarely reported.

**Results:**

Here we present the complete genomic sequence of a novel phycodnavirus, Dishui Lake Phycodnavirus 1 (DSLPV1), which was assembled from Dishui Lake metagenomic datasets. DSLPV1 harbors a linear genome of 181,035 bp in length (G + C content: 52.7%), with 227 predicted genes and 2 tRNA encoding regions. Both comparative genomic and phylogenetic analyses indicate that the freshwater algal virus DSLPV1 is closely related to the members in *Prasinovirus*, a group of marine algae infecting viruses. In addition, a complete eukaryotic histone H3 variant was identified in the genome of DSLPV1, which is firstly detected in phycodnaviruses and contributes to understand the interaction between algal virus and its eukaryotic hosts.

**Conclusion:**

It is in a freshwater ecosystem that a novel *Prasinovirus*-related viral complete genomic sequence is discovered, which sheds new light on the evolution and diversity of the algae infecting *Phycodnaviridae*.

**Electronic supplementary material:**

The online version of this article (10.1186/s12864-018-4432-4) contains supplementary material, which is available to authorized users.

## Background

The phycodnaviruses comprise genetically diverse, morphologically similar, large icosahedral (100 ~ 200 nm), and double strand DNA (180 ~ 560 kbp) viruses that infect eukaryotic algae from both fresh and marine waters, and presently contain six genera: *Chlorovirus*, *Prasinovirus*, *Prymnesiovirus*, *Phaeovirus*, *Coccolithovirus* and *Raphidovirus* [1]. The family of *Phycodnaviridae* is placed within a major, monophyletic assemblage of large eukaryotic DNA viruses, termed the nucleo-cytoplasmic large DNA viruses (NCLDVs), which was recently proposed to group into a new viral order *Megavirales* [2, 3]. Accumulating evidence suggests that these algae-infecting large viruses are active players in the aquatic ecosystem [4–6].

The hosts of phycodnaviruses are abundant and widespread in the natural environments, while almost all phycodnaviruses, including members of the *Coccolithovirus*, *Phaeovirus*, *Prasinovirus*, *Prymnesiovirus* and *Raphidovirus* genera, infect marine algae except for chloroviruses (*Chlorovirus*) that target freshwater algae [7, 8]. The members of *Prasinovirus* are the most studied among these marine algae-infecting viruses because the hosts of prasinoviruses are worldwide distributed and play a central role in the oceanic carbon cycle [9]. All known prasinoviruses infect marine photosynthetic picoeukaryotic algae in the class *Mamiellophyceae*, which mainly contains three genera: *Ostreococcus*, *Micromonas* and *Bathycoccus* [10, 11]. Thus far, several prasinoviruses have been isolated and sequenced. For instance, the *Ostreococcus* virus OtV5 (*Ostreococcus tauri* virus 5), possessing 186,234 bp long linear genome, was isolated from the green alga *O. tauri* [12], and the *Micromonas* virus MpV1 (*Micromonas* sp. RCC1109 virus 1) was isolated from *M.* sp*.* RCC1109 in eutrophic northwestern Mediterranean coastal lagoons with a genome size of 184,095 bp [13]. Interestingly, in our previous work of culture-independent metagenomic analyses, genomes of prasinoviruses-related viruses, Yellowstone Lake Phycodnaviruses (YSLPVs), were discovered in Yellowstone Lake [14], a freshwater lake in Yellowstone National Park, Wyoming.

In this study, we assemble the complete genome of a *Prasinovirus*-related novel large phycodnavirus from freshwater metagenomic datasets in Shanghai, China. Comparative genomic and phylogenetic analyses reveal unique features of this new member of *Phycodnaviridae* and shed light on the evolution, diversity and distribution of phycodnaviruses*.*

## Methods

### Metagenomic data source

The Dishui Lake (DSL) metagenomic datasets were generated with Illumina Miseq sequencer and subjected to a series of processes for quality controlling as described previously [15]. In brief, water samples were taken from neritic area of Dishui Lake (121^°^55′27.00′′ N 30^°^53′56.00′′ E) in 2013. Microbial biomasses were collected onto 0.22-μm membrane filters, from which over 6 μg genomic DNA was extracted and used for metagenomic sequencing. Raw reads were firstly analyzed with FastQC and NGS QC Toolkit prior to de novo sequence assembly. Detail information of the datasets was shown in Additional file 1: Table S1.

### Metagenomic sequence assembly of viral genome

All high-throughput sequenced reads in the DSL metagenomic paired-end libraries were assembled into contigs by using Newbler v2.6 (Roche) with default parameters. Among 1949 contigs with the length of more than 10 kb, one was initially confirmed to be related to algal large viruses with BLASTP searching of NCBI nr database and subsequently used as the template in reference assembly as previously described [16]. Briefly, this contig was used as the reference sequence to which all reads in the DSL metagenomic paired-end datasets were assembled with a minimum overlap length of 25 bp and minimum overlap identity of 95%. The reference assembly was repeated until the assembled sequence stopped extending. All assemblies were performed with the bioinformatics software Geneious R9 (Biomatters, https://www.geneious.com).

### Genome analysis

Geneious R9 software (Biomatters) was used to predict open reading frames (ORFs) by defining a start codon of ATG and a minimum 150 bp. Translated amino acid sequences were used to search (E-value < 10^−3^) for homologs in NCBI nr database by using the BLASTP program. One top hit to virus and/or non-virus was recorded. Functional annotation of ORFs was performed using the InterProScan program (http://www.ebi.ac.uk/Tools/pfa/iprscan/) [17], and conserved domain was determined with both the NCBI server and the HHpred server (http://toolkit.tuebingen.mpg.de/hhpred) [18]. Transfer RNA (tRNA) sequences were identified by using the tRNAscan-SE tool [19]. Repetitive sequences were detected with both the Geneious Pro software and the Reputer program (http://bibiserv.techfak.uni-bielefeld.de/reputer/) [20]. Whole genome alignment at the nucleotide level was performed with the MAUVE software [21].

### Phylogenetic analysis

Maximum likelihood phylogenetic trees were reconstructed based on homologs of DNA polymerase B family (PolB) gene, nicotinamide adenine dinucleotide (NAD)-dependent epimerase/dehydratase gene and histone H3 gene, respectively. Homolog counterparts from a range of representative viruses, bacteria and eukaryotes were downloaded from the NCBI protein database (https://www.ncbi.nlm.nih.gov/protein). Amino acid sequences were multiply aligned with the MUSCLE program, followed by tree reconstruction with the MEGA 7 software by using the JTT model and bootstrap value of 100 [22].

### PCR for the histone gene

To avoid any possibilities of the misassembly of the histone gene in the assembled viral genome, a pair of PCR primers (5’-CTTGAGTATGGCACCCTTGG-3′ and 5’-TCGCTTGGCGTCTTTCAAGG-3′) respectively targeting the up- and downstream of the histone gene were designed based on the assembled vial genomic sequence. PCR was then performed with the same DNA samples as applied to the metagenomic sequencing. The reaction (25 μL) contained 0.4 mM of forward and reverse primers, 12.5 μL of *Taq* PCR master mix (2×) (Tiangen Biotech), and 20–25 ng of DNA. The amplification started with the program: initial denaturation at 94 °C for 4 min, followed by 30 cycles of 94 °C for 30 s, 58 °C for 30 s, and 72 °C for 45 s in an Eppendorf Mastercycler machine. PCR products were cloned and sequenced as previously described [15]. Sequences were aligned to the assembled genome by using Geneious R9 (Biomatters, https://www.geneious.com).

### Homology modeling of Histone H3 in DSLPV1

The three dimensional structure of histone fold H3 in DSLPV1_013 was modeled on the online protein homology modeling SWISS-MODEL server (www.swissmodel.expasy.org) with the template of the crystal structure of human histone H3 (PDB id 3lel.1.E, amino acid sequence identity 83.82%). The structure was visualized and analyzed with the PyMOL software (www.pymol.org) [23].

### NCLDV conserved genes and virophage related genes in DSLPV1

The nucleo-cytoplasmic virus orthologous group proteins (NCVOG) [24] were downloaded from NCBI (ftp://ftp.ncbi.nih.gov/pub/wolf/COGs/NCVOG) for establishing a local database by using ‘make blastdb’ command in a BioLinux server. Similarly, all predicted proteins of Dishui Lake Virophage 1 (DSLV1, accession number KT894027) were downloaded to construct another local dataset. Predicted proteins of DSLPV1 were used as queries to search these two datasets respectively by using BLASTP program with cutoff e-value of 1e-3. The output results were recorded and analyzed for the NCLDV conserved genes and virophage related genes in DSLPV1.

### Nucleotide sequence accession number

The DSLPV1 sequence has been deposited in the GenBank database (accession no. KY747489).

## Results

### General organization of the DSLPV1 genome

**Fig. 1:**
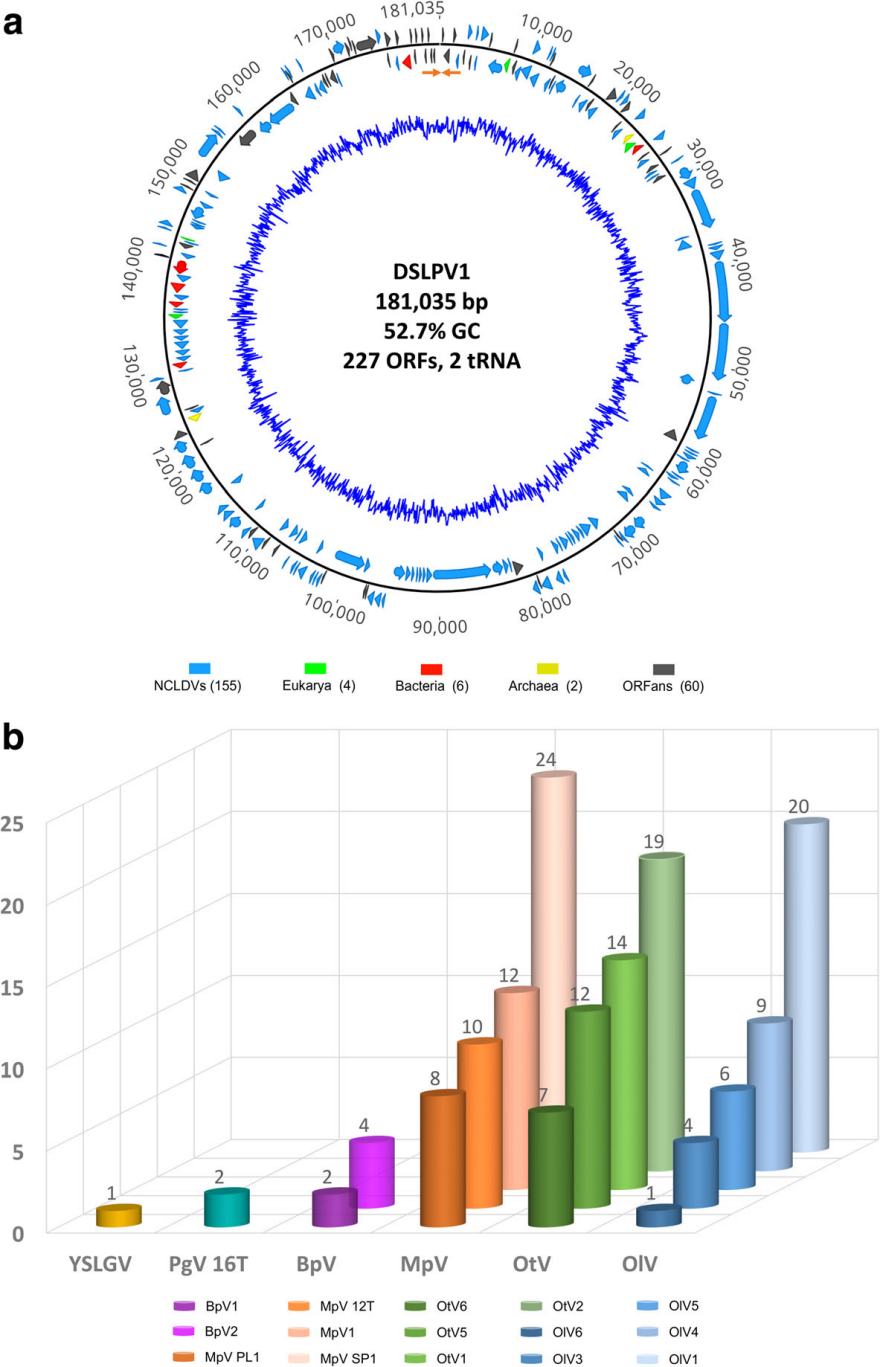
Features of the DSLPV1 genome. **a** Genomic map of DSLPV1. The linear genome of DSLPV1 is shown in an open circle. The terminal inverted repeats of the linear genome are indicated with orange arrows. The outside numbers represent the position of nucleotide. ORFs are indicated with box arrows in different colors, which represent different taxon categories of the ORFs (forward and reverse strands respectively): light blue for NCLDVs hits (*n* = 155), light green for eukaryote hits (*n* = 4), red for bacteria hits (*n* = 6), yellow for archaea hits (*n* = 2) and gray for ORFans (ORFs with no BLAST hits in public databases, *n* = 60). The inner blue line represents G + C content. Viral name, genomic length, G + C content and the total number of predicted ORFs and the number of tRNA are showed in the center. **b** The number of top BLASTP hits of the DSLPV1 ORFs to large/giant viruses that infect algae. Different species are indicated in different colors. The full name of each virus is shown as following: BpV- *Bathycoccus* sp. RCC1105 virus; MpV- *Micromonas pusilla* virus; OlV- *Ostreococcus lucimarinus* virus; OtV- *Ostreococcus tauri* virus; PgV 16 T- *Phaeocystis globosa* virus 16 T; YSLGV- Yellowstone lake giant virus

After the reference assembly, more than 8000 reads were eventually mapped to the consensus sequence (the contig that was initially confirmed to be related to algal large viruses, which is described in “Metagenomic sequence assembly of viral genome” in Methods.) with a high coverage (average coverage = 78.7, Additional file 1: Table S2) across the whole genome (Additional file 1: Figure S1), which indicated the accuracy of sequence assembly. The obtained genomic sequence with a size of 181,035 bp was named that of Dishui Lake Phycodnavirus 1 (DSLPV1). A 402-bp terminal inverted repeat was identified at both ends of the genome (Fig. 1a), which indicates the complete and linear genome of the DSLPV1. The G + C content of the genome is 52.7%, which is much higher than that of prasinoviruses (37.0–44.6%) but resembles YSLPVs (47.7–55.0%). A total of 227 ORFs were predicted, which are evenly distributed on both positive and negative DNA strands through the genome (i.e. 48% on the positive strand) with an average gene length of 770 bp and a coding density of 1.254 genes per kbp.

As the results of BLASTP search against NCBI nr database showed (Additional file 1: Table S3), among these 227 ORFs, 167 (~74%) shared similarity with proteins in nr database, with 155 having the top hits to algae large/giant viruses in NCLDVs, 6 to bacteria, 4 to eukaryotes and 2 to archaea (Fig. 1 and Additional file 1: Table S3). Among the remaining 60 ORFs (~26%) having no hits in NCBI nr database (Fig. 1a), 35 had hits to NCBI environmental database (env_nr). Interestingly, 98% (152 of 155) ORFs having the best matches to giant/large viruses were homologous to algal large viruses in the *Prasinovirus* genus of the *Phycodnaviridae* family (Fig. 1b). Meanwhile, 75 out of these 155 ORFs with virus-hits showed no BLAST hits to cellular life (Additional file 1: Table S3), which are considered as virus specific genes, and the rest of 80 ORFs had both virus and non-virus hits (Additional file 1: Table S3). Of the four eukaryote-derived ORFs, one showed the best match to *Ostreococcus lucimarinus* CCE9901, the natural host of prasinoviruses, which indicates the potential genetic links between algae host and DSLPV1. In addition, two regions encoding tRNA (one Gln-tRNA and one Asn-tRNA) were identified in the DSLPV1 genome.

### Gene annotation in the DSLPV1 genome

Functional annotation analysis of 227 putative proteins in DSLPV1 indicated that 160 (70.5%) had no homologues with defined functions in public databases. The remaining 67 putative proteins (29.5%) with annotated functions were classified into nine functional categories (Table 1). Ten putative proteins are involved in DNA replication, recombination and repair, which imply the independence of viral DNA replication in host cell; three putative proteins are associated with nucleotide transport and metabolism, eight with transcription, eight with sugar manipulation, three with DNA restriction/methylation, and eight with protein and lipid synthesis/modification; seven encode capsid proteins; three have other miscellaneous functions, and one possibly possesses a signaling function.

**Table 1:** DSLPV1 ORFs with annotated functions

Function and protein	ORF
DNA, RNA, replication, recombination and repair	
DNA polymerase	189
DNA topoisomerase I	133
DNA topoisomerase II	195
PCNA	96
Exonuclease	115
RNase H	142
SF3 helicase	152
NTPase/helicase	104
VV A18 helicase	52
DNA polymerase III epsilon subunit	209
Nucleotide transport and metabolism	
Ribonucleotide reductase, large subunit	119
Ribonucleotide reductase, small subunit	139
ATPase (VV A32 virion packaging ATPase)	80
Transcription	
mRNA capping enzyme	77
RNase III	114
TATA-box binding protein	125
Poxvirus late transcription factor VLTF3	98
Nudix hydrolase	68
Transcription elongation factor S-II	178
Transcription factor MYB1R1	163
Transcription initiation factor IIB	162
Sugar manipulation enzymes	
Mannosyltransferase	170
N-acetylglucosamine transferase	42
Glycosyltransferase	43
Glycosyltransferase	62
Glycosyltransferase	166
Glycosyltransferase	169
Glycosyltransferase	200
NAD-dependent epimerase/dehydratase	41
DNA restriction/methylation	
Adenine-specific methyltransferase	10
DNA-cytosine methyltransferase	149
Methyltransferase FkbM	100
Protein and lipid synthesis/modification	
Prolyl 4-hydroxylase alpha-subunit	103
Ubiquitin C-terminal hydrolase	158
ATP-dependent protease proteolytic subunit	16
FtsH2 metalloprotease	12
33 kDa in vitro translation peptide	118
N-myristoyltransferase	76
Patatin phospholipase	106
Asparagine synthetase	181
Capsid	
Capsid protein 1	53
Capsid protein 2	59
Capsid protein 3	81
Capsid protein 4	82
Capsid protein 5	147
Capsid protein 6	188
Capsid protein 7	194
Miscellaneous	
Virus inclusion body	105
Rhodanese domain-containing protein	217
Fibronectin-binding protein	8
Signaling	
PhoH	183

ORF numbers refer to that of DSLPV1. Their GenBank annotations are shown in Additional file 1: Table S3

### NCLDV conserved genes in the DSLPV1 genome

DSLPV1 contains a nearly complete set of conserved genes in NCLDVs (40/47), including core genes of DNA polymerase B family protein, A18 helicase, A32 virion packaging ATPase and seven copies of major capsid protein. Ten NCVOGs in prasinoviruses were present in DSLPV1, and other key feature genes in prasinoviruses were also detected in DSLPV1 (Table 2). Notably, although some core genes were undetected in DSLVP1, e.g., serine/threonine protein kinase, they were probably functionally displaced by other genes. For instance, DSLVP1 ORF27 has a protein kinase structure which could be the functional substitute of the serine/threonine protein kinase. Such varied genomic features distinguish DSLPV1 from the prasinoviruses.

**Table 2:** Conserved genes present in DSLPV1 and the prasinoviruses

	Gene name	OtV5	MpV1	BpV1	BpV2	DSLPV1
NCLDV core gene	DNA polymerase	1	1	1	1	1
	ATPase (DNA packaging)	1	1	1	1	1
	A18 helicase	1	1	1	1	1
	Capsid protein	8	8	7	7	7
	Serine/Threonine protein kinase	1	1	1	1	0
NCVOGs present in prasinoviruses	Poxvirus late transcription factor VLTF3	1	1	1	1	1
	DNA topoisomerase	2	1	1	1	2
	Transcription factor IIS	1	1	1	1	1
	Transcription factor IIB	1	1	1	1	1
	Ribonucleotide reductase small subunit	1	1	1	1	1
	Ribonucleotide diphosphate reductase large subunit	1	1	1	1	1
	mRNA capping enzyme	2	2	2	2	2
	ATP dependant DNA ligase	1	1	1	1	0
	Exonuclease (YqaJ)	2	2	2	2	1
	dUTP pyrophosphatase	1	1	1	1	0
	Thymidine kinase	1	1	1	1	0
	Thymidylate S	1	1	1	1	0
	Nudix hydrolase	1	1	1	0	1
	RuvC, Holliday junction resolvases, Extended Pox_A22, Poxvirus A22 family	2	2	1	1	2
Other prasinovirus common genes	PCNA	1	1	1	1	1
	NTPase/helicase	1	1	1	1	1
	RNase H	1	1	1	1	1
	RNase III	1	1	1	1	1
	Transcription activator /SWI/SNF helicase	1	1	1	1	0
	TATA-box binding protein (TBP)	1	1	1	1	1
	Prolyl 4-hydroxylase	1	1	1	1	1
	33 kDa in vitro translation peptide	2	3	2	2	2
	2OG-Fe(II) oxygenase	1	1	1	1	1
	Aspartyl/Asparaginyl beta-hydrolase	1	1	1	1	0
	Ubiquitin hydrolase-like cystein peptidase	1	1	1	1	1
	FtsH (3–4-24) metalloendopeptidase	1	1	1	1	1
	ATP-dependant protease proteolytic subunit	1	1	1	1	1
	Patatin-like phospholipase	1	1	1	1	1
	Potassium channel protein, PhoH	1	1	1	1	1
	ABC-1 domain protein	1	1	1	1	1
	ATP/GTP binding site motif A AGB-1	1	1	1	1	1
	Viral A-type inclusion protein	1	1	1	1	1

The numbers in the table represent that of gene copies

### Genomic architecture of DSLPV1

**Fig. 2:**
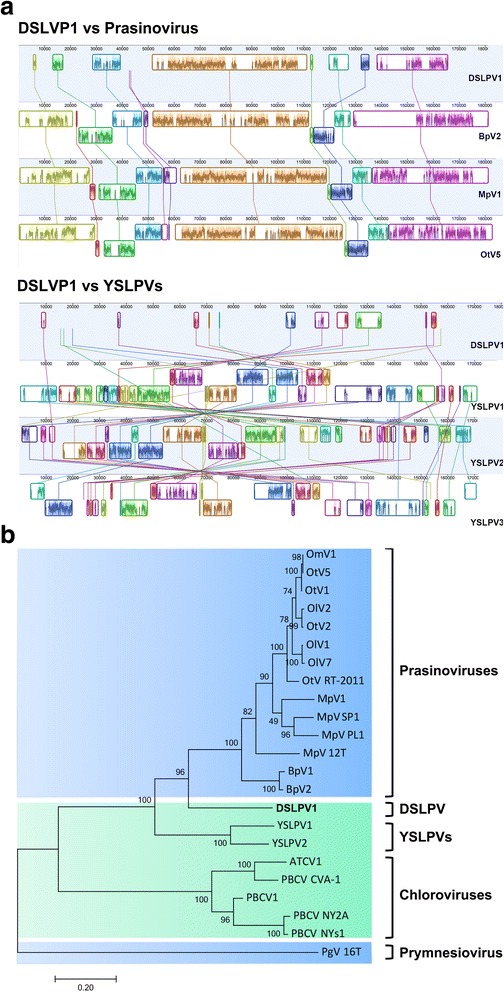
Relationship of DSLPV1 to the phycodnaviruses. **a** Whole genome alignment of DSLPV1 with the representative prasinoviruses and the YSLPVs. Different conserved regions shared among the genomes are indicated in different colors. **b** Maximum-likelihood phylogenetic tree of the B-family DNA polymerase (PolB) proteins. Marine viruses are shadowed in light blue, and freshwater viruses are shadowed in light green. Different families/groups of viruses are labeled with black lines and names on the right of the tree. The scale bar indicates a distance of 0.2 fixed mutations per amino acid position. GenBank accession number of the PolB sequences used for this tree is listed in Additional file 1: Table S4. OmV: *Ostreococcus mediterraneus* virus; OtV: *Ostreococcus tauri* virus; OlV: *Ostreococcus lucimarinus* virus; MpV: *Micromonas pusilla* virus; BpV: *Bathycoccus* sp. RCC1105 virus; DSLPV: Dishui Lake Phycodnavirus; YSLPV: Yellowstone Lake phycodnavirus; ATCV: *Acanthocystis turfacea* Chlorella virus; PBCV: *Paramecium bursaria* Chlorella virus; PgV: *Phaeocystis globosa* virus

Whole genome of DSLPV1 was aligned with that of the prasinoviruses (OtV5, MpV1, BpV2) and YSLPV1–3, respectively (Fig. 2a). DSLPV1 revealed good sequence synteny with the prasinoviruses, but shared few homologous segments with the YSLPVs. In addition, the gene content analysis also showed that DSLPV1 shared more homologous genes with these three prasinoviruses (OtV5: 59%, MpV1: 58%, BpV2: 61%) than with YSLPV1 (42%). These results suggest the DSLPV1 genomic architecture is more analogous to the prasinoviruses than to the YSLPVs.

### Phylogenetic affiliation of DSLPV1

The annotated DNA polymerase gene (DSLPV1_189) of DSLPV1 shared high amino acid similarity (52–60%) with that of other phycodnaviruses, especially the prasinoviruses and the YSLPVs. Phylogenetic analysis of this gene and its homologues in other giant/large viruses indicated that DSLPV1 formed a monophyletic group with *Prasinovirus* with robust bootstrap support, and both DSLPV1 and the prasinoviruses are the sister lineages to the YSLPVs (Fig. 2b).

### Host like genes in the DSLPV1 genome

A total of 44 ORFs in the DSLPV1 genome showed amino acid similarity (coverage > 70%, identity > 25%) to genes found in the algae from both freshwater and marine environments, especially 12 ORFs that share high amino acid similarity (30–87%) to genes in the *Mamiellophyceae* class (green algae), containing natural hosts of the prasinoviruses (Table 3). Meanwhile, DSLPV1_139, the putative ribonucleoside-diphosphate reductase small subunit gene (*rnr2*) that is often shared by the prasinoviruses and their algae hosts, showed high amino acid similarity (63%) to the *rnr2* in *Ostreococcus lucimarinus* CCE9901 (Table 3). In addition, DSLPV1_041 encodes a putative NAD-dependent epimerase/dehydratase protein that appears to derive from *Ostreococcus lucimarinus* CCE9901 as well (Table 3, Additional file 1: Figure S2).

**Table 3:** Host like genes in DSLPV1

ORF	Putative function	Hit	Identity (%)	Coverage (%)	E value
DSLPV1_008	Fibronectin-binding protein	OL	38	95	8.00E-11
DSLPV1_013	Histone H3	OL	87	71	1.00E-78
DSLPV1_041	NAD-dependent epimerase	OL	48	97	3.00E-89
DSLPV1_043	Glycosyltransferase	OT	27	69	5.00E-09
DSLPV1_076	N-myristoyltransferase	MP	36	99	1.00E-48
DSLPV1_096	PCNA	MP	30	97	3.00E-31
DSLPV1_113	DNA adenine methyltransferase	Ms	33	93	2.00E-08
DSLPV1_139	Ribonucleotide reductase, ss	OL	63	73	2.00E-135
DSLPV1_159	mRNA-capping enzyme	OT	26	93	5.00E-24
DSLPV1_164	Unknown	MP	46	96	3.00E-18
DSLPV1_178	Transcription factor SII	OL	33	98	8.00E-12
DSLPV1_195	DNA topoisomerase II	OT	43	100	0

*OL Ostreococcus lucimarinus* CCE9901, *OT Ostreococcus tauri*, *MP Micromonas pusilla* CCMP1545, *Ms. Micromonas* sp. RCC299

### Complete eukaryotic histone fold H3 in the DSLPV1 genome

**Fig. 3:**
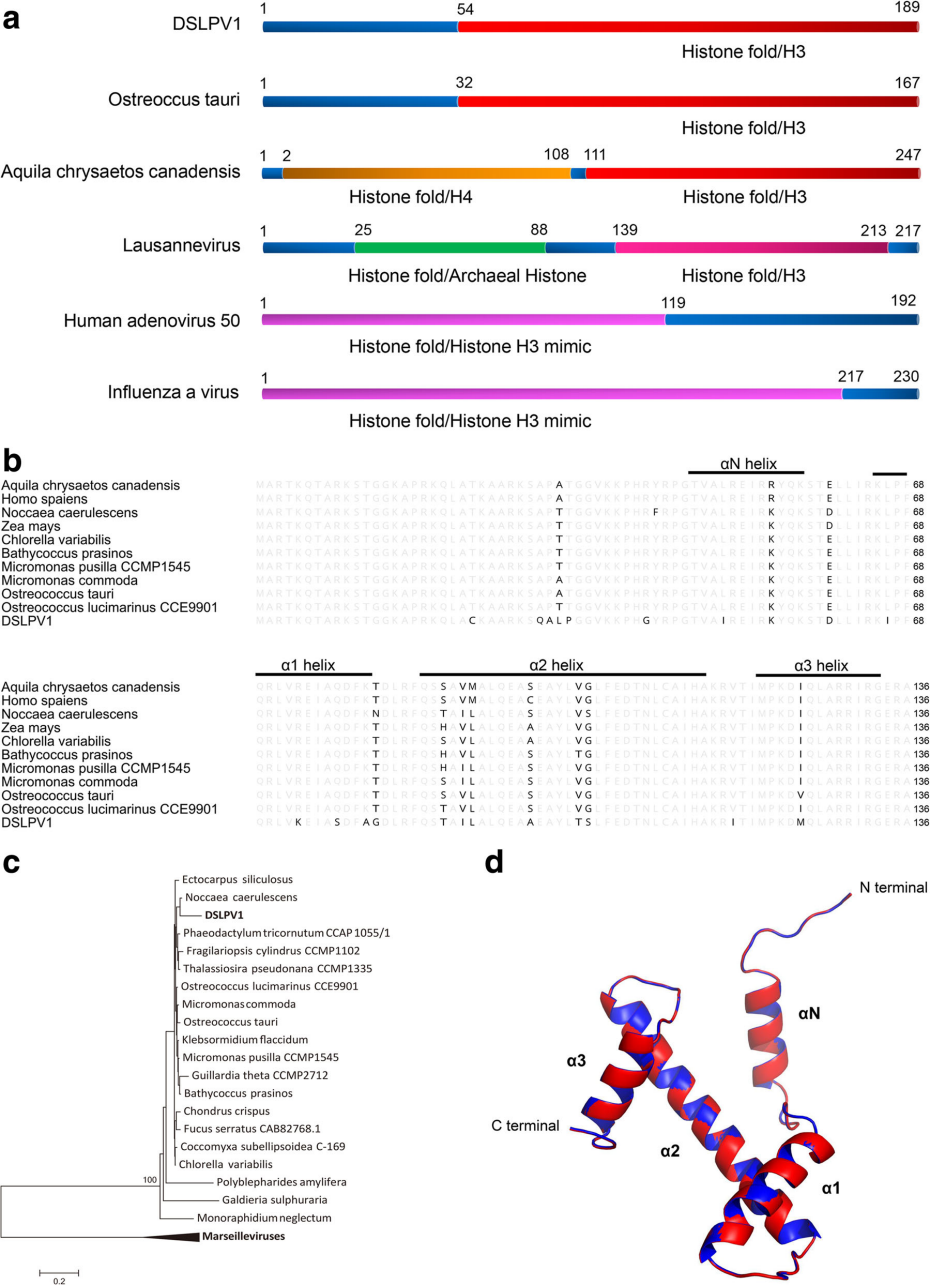
Viral histone H3 gene in DSLPV1. **a** Schematic of viral histone H3 in DSLPV1 and its relatives in representative eukaryotes and other viruses. Different histone fold regions are marked in different colors, and the similar histones are indicated in the same color, i.e., eukaryotes derived histones in red, viral histones in pink and histone mimics in purple. **b** Amino acid sequence alignments of the DSLPV1 histone fold H3 and the representative eukaryotic histone H3 folds. Diverged amino acids are emphatically visualized and canonical helixes of histone fold are indicated. **c** Maximum likelihood phylogenetic tree of the viral and the representative eukaryotic histone fold H3 proteins. GenBank accession numbers of the histone sequences are listed in Additional file 1: Table S4. Only more than 50% of bootstrap value is shown in the tree. **d** Predicted three dimensional structure of the histone H3 fold in DSLPV1. The protein template used for the prediction is shown in blue and the target protein is in red

Interestingly, DSLPV1 harbored a histone like gene (DSLPV1_013), which had high amino acid similarity (74–88%) to the histone gene from a wide range of eukaryotes. To eliminate the possibility of metagenomic sequence misassembly, the histone gene of DSLPV1_013 was re-checked with the DSLPV1 specific PCR and sequencing analysis. Sequence alignment analysis revealed that mismatches of the histone gene were not observed between the PCR amplified sequence and the assembled genome, which confirms the presence of a histone like gene in DSLPV1. Protein fold recognition with the InterProScan software detected a complete histone fold H3 at the C terminal of this protein (Fig. 3a). Moreover, sequence comparison of representative histone H3 folds from eukaryotes, selected based on BLASTP results, with that of DSLPV1 revealed high conservation between them (Fig. 3b), and the phylogenetic analysis indicates that the histone gene of DSLPV1 is closely related to that of eukaryotes but not viruses (Fig. 3c). Meanwhile, the predicted structure of DSLPV1 histone fold H3 revealed a canonical eukaryotic histone fold (Fig. 3d). In addition, the G + C content of the DSLPV1 histone gene (57%) is incongruous with that of the DSLPV1 genome (52.7%) but coherent with that of some algal genomes [25, 26]. Taken together, these results indicate the possibility of a recent horizontal transfer of the histone gene from eukaryotic hosts, e.g., algae, to viruses.

### Genetic links between DSLPV1 and DSLV1

Intriguingly, three homologous genes that are shared between DSLPV1 and Dishui Lake Virophage 1 (DSLV1) were detected based on BLAST when searching the local database that consists of all predicted DSLV1 proteins by using the baits of 227 ORFs of DSLPV1. Functional annotation of these three homologous proteins revealed that the DSLV1_02 harbored two transmembrane regions at N terminal, which were analogous to DSLPV1_019 (26.6% of amino acid similarity) and DSLPV1_141 (25.4% of amino acid similarity). Similar RING-type zinc finger domain was detected at C terminal of DSLV1_25 and DSLPV1_175 (29.4% of amino acid similarity), and this domain is probably involved in mediating protein-protein interactions. In addition, a collagen triple helix repeat domain at C terminal of DSLV1_09 showed 41.2% of amino acid identity to DSLPV1_201, which represents the highest sequence similarity in comparison with the similarity that was shared between the other homologies described above.

## Discussion

In this study, the genomic sequence of a novel phycodnavirus, named DSLPV1, was obtained from the DSL metagenomic datasets. The DSLPV1 genome appears to be complete as it contains a pair of 402-bp terminal inverted repeats flanking both ends of the linear genome as well as a nearly complete set of conserved genes of NCVOGs. Both comparative genomic and phylogenetic analyses indicate that DSLPV1 is closely affiliated to the prasinoviruses since they are similar in genomic length, share the most number of homologous genes including almost complete set of the *Prasinovirus* conserved genes (27/32), and formed a monophyletic clade based on the DNA PolB protein phylogeny. Accordingly, DSLPV1 currently represents the closest freshwater relative of the marine prasinoviruses.

Dishui Lake where DSLPV1 was discovered is a holomictic freshwater lake (0.8‰ of salt) that was artificially excavated on tidal flat in 2003. Freshwater from neighboring Dazhi River is diverted into Dishui Lake, which is regularly discharged to the East China Sea. Interestingly, DSLPV1 is closely affiliated with the *Prasinovirus* infecting marine algae in the *Mamiellophyceae* class rather than with the freshwater-originated *Prasinovirus*-related YSLPVs as well as with the members in the *Chlorovirus* genus that are presently the only well-defined freshwater algae viruses in *Phycodnaviridae* [27].

Viruses that are closely related to the prasinoviruses may have the *Mamiellophyceae* algae like host since all known prasinoviruses infect the *Mamiellophyceae* algae [28–30]. Meanwhile, some evidence showed the existence of freshwater algae species in *Mamiellophyceae*, albeit no phycodnaviruses were detected [10]. We consequently speculate that the host of DSLPV1 may be some kinds of freshwater *Mamiellophyceae* algae related species. Furthermore, the closest homologues of several viral genes in DSLPV1, especially the histone H3 gene, are present in the *Ostreococcus* algae as well, e.g., *O. lucimarinus*, *O. tauri* (Table 3)*.* These genetic links between DSLPV1 and green algae not only further support the above proposed hypothesis, but also give better insight into a theoretical guidance for the isolation of potential algal host of DSLPV1. In addition, the *Mamiellophyceae* related contigs are the most abundant in the algae-related contigs that were identified in the DSL metagenomic datasets (data not shown). Hence, all these evidence indicates the possibility of the interaction relationship between DSLPV1 and the *Ostreococcus* related algae in freshwater. To further shed insight on the host of DSLPV1, both the DSLPV1 and its algae host need to be isolated, and infection experiments had to be performed. Notably, on the phylogenetic tree of the histone fold H3 protein (Fig. 3c), DSLPV1 was grouped together with the *Noccaea caerulescens* plant but not with the *Ostreococcus* algae. However, given that the bootstrap value is less than 50%, at almost all the branching points in the sub-tree comprising DSLPV1, the *Ostreococcus* algae and other representative eukaryotes, due to the high conservation of the histone fold H3 proteins, the phylogenic affiliation of the DSLPV1 histone fold H3 protein remains uncertain. Accordingly, the horizontal transfer of the histone H3 fold protein gene may occur between DSLPV1 and the *Ostreococcus* algae. It hints again the *Ostreococcus* related algae might be the host of DSLPV1.

Surprisingly, a eukaryote derived histone like gene was identified in the genome of DSLPV1, which is firstly reported in the phycodnaviruses. It has been suggested that histones that associate to form the nucleosome and wrap the DNA in eukaryotic cells were probably acquired or mocked by viruses [31, 32]. Viral histones carry largely unknown functions [31–38]. Significant roles that are played by a few viral histones were demonstrated. The histone H4 protein in insect bracoviruses that shares high sequence identity with its host gene plays a critical role in suppressing host immune responses during infection by competing with endogenous cellular H4 for incorporation into the chromatin [39]. Interestingly, the nonstructural protein 1 (NS1 protein) that is encoded by some influenza virus strains, mimicry of the histone H3, suppresses the antiviral response of host by sequestering crucial factors required for transcriptional elongation [31]. Similarly, human adenovirus protein VII mimics cellular histone H3 for binding host nucleosomes to sequester immune danger signals to evade immune system during infection [40]. Alike the histone H4 in bracoviruses, the DSLPV1 histone H3 showed high amino acid similarity (87%) to the histone gene in its potential algae host except for an extra 53-aa tail at N terminal. In addition, sharing the same modification sites in N-terminal tail and canonical helixes in core fold between DSLPV1 and the cellular histone H3 renders us to speculate the similar functions of the DSLPV1 histone. Regretfully, the host of DSLPV1 is still unknown which debilitates the study of virus-host interaction to figure out the roles of viral histone in DSLPV1.

In our previous study, the genomes of algal large novel viruses YSLPVs and Yellowstone Lake giant virus (YSLGV) were discovered in Yellowstone Lake [14]. Meanwhile, interestingly the genomes of virophage YSLVs that are considered as the giant viral parasites were found in the same lake [16, 41]. Additionally, the genomes of virophages (Organic Lake Virophage, OLV, and Qinghai Lake Virophage, QLV) and their potential giant viral hosts, e.g., mimiviruses or phycodnaviruses, were obtained in Antarctic Organic Lake and Qinghai Lake, China [6, 42]. Coincidently, in addition to DSLPV1, a novel virophage DSLV1 that is closely related to Yellowstone Lake Virophage 3 (YSLV3) in Yellowstone Lake was discovered in Dishui Lake as well [15]. Interestingly, DSLPV1 and DSLV1 share the collagen triple helix repeat containing protein that was suspected to be involved in protein-protein interaction in virophage Sputnik/host mamavirus and virophage OLV/potential host Organic Lake Phycodnavirus (OLPV) associations [6, 43]. Genetic links were also observed between the YSLPVs and the YSLVs. However, their potential associations await future exhaustively experimental study.

## Conclusions

In conclusion, a complete linear genomic sequence of DSLPV1 is discovered based on sequence assembly of the metagenomic datasets from Dishui Lake in Shanghai, China. Comprehensive genomic and phylogenetic analyses reveal that DSLPV1 represents a novel viral species in freshwater aquatic ecosystem and is closely related to the marine algae infecting *Prasinovirus* in the family *Phycodnaviridae*. Recent horizontal gene transfer was detected between DSLPV1 and its potential algal host. Our results here and the previous [14] suggest that the diversity and distribution of freshwater algal large or giant viruses remain far beyond exploration. Such knowledge will significantly contribute to better understanding not only the evolution of the phycodnaviruses and other related giant viruses, e.g., mimiviruses, but also their parasite viruses of virophages.

## Additional file

Additional file 1: Supplementary data. It contains all supplementary figures and tables. (DOCX 447 kb)

## Abbreviations

dsDNA: Double-stranded DNA
DSL: Dishui Lake
DSLPV1: Dishui Lake Phycodnavirus 1
DSLV1: Dishui Lake Virophage 1
NCLDVs: Nucleo-cytoplasmic large DNA viruses
NCVOG: Nucleo-Cytoplasmic Virus Orthologous Group
nr database: Non-redundant database
NS1 protein: Non-Structural protein 1
OLPV: Organic Lake Phycodnavirus
OLV: Organic Lake Virophage
ORF: Open Reading Frame
PCR: Polymerase Chain Reaction
QLV: Qinghai Lake Virophage
rnr2: ribonucleoside-diphosphate reductase small subunit
YSLGV: Yellowstone Lake giant virus
YSLPVs: Yellowstone Lake Phycodnaviruses
YSLV: Yellowstone Lake Virophage
