# Supplementary material for: The genome of a prasinoviruses-related freshwater virus reveals unusual diversity of phycodnaviruses
Source: BMC Genomics. 2018 Jan 15;19:49. doi: 10.1186/s12864-018-4432-4 (PMC5769502; doi:10.1186/s12864-018-4432-4)
Supplement: Additional file 1: — Supplementary data. It contains all supplementary figures and tables. (DOCX 447 kb) [file 12864_2018_4432_MOESM1_ESM.docx]

**Additional file**

**The genome of a prasinoviruses-related freshwater virus reveals unusual diversity of phycodnaviruses**

Hao Chen^a^, Weijia Zhang^a,#^, Xiefei Li^a^, Yingjie Pan^a,b^,

Shuling Yan^a,c^, Yongjie Wang^a,b,d*^

^a^ College of Food Science and Technology, Shanghai Ocean University, Shanghai, China;

^b^ Laboratory of Quality and Safety Risk Assessment for Aquatic Products on Storage and Preservation, Ministry of Agriculture, Shanghai, China;

^c^ Institute of Biochemistry and Molecular Cell Biology, University of Göttingen, Göttingen, Germany;

^d^ Laboratory for Marine Biology and Biotechnology, Qingdao National Laboratory for Marine Science and Technology, Qingdao, China.

Running title: **Genome of DSLPV1**

^*^Corresponding author: Tel.: +86 21 61900505; email: yjwang@shou.edu.cn.

^#^Present address: Archaea Center, Department of Biology, Copenhagen University, DK2000 Copenhagen N, Denmark

**Table S1** Information of the DSL Metagenomic datasets

| **Run** | **Raw data (reads)** | **QC by the pipeline (reads)** | **QC by NGS (reads)** |
| --- | --- | --- | --- |
|  |  |  |  |
| 1 | 35,371,138 | 23,505,862 | 23,151,200 |
| 2 | 20,766,464 | 17,580,296 | 17,507,722 |
| 3 | 7,974,858 | 6,239,280 | 6,202,911 |
| 4 | 17,264,916 | 14,037,596 | 13,964,065 |
| 5 | 15,136,922 | 12,272,954 | 12,216,490 |
| 6 | 1,833,418 | 1,407,000 | 1,387,258 |

| **Name** | **No. of reads mapped to genome** | **No. of identical sites** | **Pairwise Identity (%)** | **Genome coverage** | | | **Size of dataset (Gb)** |
| --- | --- | --- | --- | --- | --- | --- | --- |
|  |  |  |  | **Mean** | **Minimum** | **Maximum** |  |
| DSLPV1 | 83,791 | 151,093 | 99.4 | 78.7 | 16 | 141 | 22.48 |
|  |  |  |  |  |  |  |  |

**Table S2** Metagenomic assembly information of the DSLPV1 genome

**Table S3** Annotated ORFs of the DSLPV1 genome

| **ORF #** | **aa length** | **Best BLASTP hit in GenBank nr database** | | | | **Conserved Domain (E-value; identifier; alignment position [start-end])** |
| --- | --- | --- | --- | --- | --- | --- |
|  |  | **Accession number** | **Best hits in nr database** | **E-value** | **Identity %** |  |
| **4** | **132** | **AFC35123.1** | **hypothetical protein OtV6_215c [Ostreococcus tauri virus RT-2011]** | **2.00E-49** | **58** |  |
| 6 | 189 | YP_001648280.1 | hypothetical protein OsV5_204r [Ostreococcus virus OsV5] | 1.00E-76 | 60 | HAD_like superfamily (8.88E-04; cl21460; 3-143) |
| 8 | 127 | YP_007676282.1 | hypothetical protein MPVG_00218 [Micromonas pusilla virus 12T] | 2.00E-25 | 48 | DUF814 superfamily (1.61E-16; cl05307; 5-89) |
| **9** | **70** | **YP_004061791.1** | **hypothetical protein OlV1_159 [Ostreococcus lucimarinus virus 1]** | **8.00E-17** | **59** |  |
| 10 | 269 | YP_008052748.1 | adenine specific DNA methyltransferase [Phaeocystis globosa virus] | 1.00E-104 | 57 | N6_N4_Mtase (4.11E-26; pfam01555; 85-262) |
| 12 | 571 | AET84990.1 | cell division protein [Micromonas pusilla virus SP1] | 0 | 60 | AAA (2.04E-23; cd00009; 149-314) |
| 13 | 189 | XP_011598903.1 | PREDICTED: histone H3-like [Aquila chrysaetos canadensis] | 8.00E-81 | 74 | H2A superfamily (1.21E-48; cl00074; 54-186) |
| 16 | 194 | AFC35096.1 | hypothetical protein OtV6_188c [Ostreococcus tauri virus RT-2011] | 4.00E-65 | 52 | crotonase-like superfamily (7.76E-25; cl21466; 25-187) |
| **17** | **370** | **YP_004061816.1** | **hypothetical protein OlV1_184c [Ostreococcus lucimarinus virus 1]** | **2.00E-99** | **44** | **AAA_16 (1.27E-03; pfam13191; 24-74)** |
| **18** | **236** | **YP_001648287.1** | **hypothetical protein OsV5_211f [Ostreococcus virus OsV5]** | **1.00E-11** | **44** |  |
| 19 | 288 | ADQ91345.1 | hypothetical protein BpV2_178c [Bathycoccus sp. RCC1105 virus BpV2] | 9.00E-72 | 51 |  |
| **20** | **118** | **YP_001648289.1** | **hypothetical protein OsV5_213f [Ostreococcus virus OsV5]** | **1.00E-42** | **58** |  |
| **21** | **101** | **AET84939.1** | **hypothetical protein MPXG_00141 [Micromonas pusilla virus SP1]** | **3.00E-13** | **46** |  |
| 23 | 156 | AET84910.1 | hypothetical protein MPXG_00112 [Micromonas pusilla virus SP1] | 9.00E-53 | 54 |  |
| **25** | **121** | **AET43739.1** | **hypothetical protein MPWG_00255 [Micromonas pusilla virus PL1]** | **5.00E-31** | **50** |  |
| **26** | **431** | **YP_001648142.1** | **hypothetical protein OsV5_065f [Ostreococcus virus OsV5]** | **1.00E-152** | **52** | **NAD_binding_8 (2.36E-06; pfam13450; 7-69)** |
| 27 | 497 | YP_003212883.1 | hypothetical protein OTV1_059 [Ostreococcus tauri virus 1] | 0 | 59 | ABC1_ADCK3-like (3.78E-84; cd05121; 82-312): PKc_like superfamily (3.75E-84; cl21453; 82-312) |
| **28** | **87** | **YP_003212881.1** | **hypothetical protein OTV1_057 [Ostreococcus tauri virus 1]** | **5.00E-09** | **42** | **Lipocalin superfamily (5.09E-03; cl21528; 22-82)** |
| 30 | 239 | ADQ91213.1 | hypothetical protein BpV2_046 [Bathycoccus sp. RCC1105 virus BpV2] | 3.00E-110 | 65 | Pox_A22 superfamily (4.90E-10; cl04798; 1-161) |
| 32 | 288 | ADQ91212.1 | hypothetical protein BpV2_045 [Bathycoccus sp. RCC1105 virus BpV2] | 4.00E-41 | 36 |  |
| 34 | 140 | YP_007674696.1 | hypothetical protein OLNG_00052 [Ostreococcus lucimarinus virus OlV5] | 1.00E-52 | 60 |  |
| 35 | 179 | AET43729.1 | hypothetical protein MPWG_00245 [Micromonas pusilla virus PL1] | 1.00E-61 | 54 | zf-FCS (4.04E-03; pfam06467; 3-43) |
| **38** | **223** | **AFC34951.1** | **hypothetical protein OtV6_043 [Ostreococcus tauri virus RT-2011]** | **7.00E-37** | **48** |  |
| **39** | **240** | **YP_007674859.1** | **hypothetical protein OLNG_00221 [Ostreococcus lucimarinus virus OlV5]** | **5.00E-40** | **36** | **O-FucT_like superfamily (2.57E-10; cl16914; 86-233)** |
| 40 | 199 | WP_048118396.1 | hypothetical protein [Candidatus Nitrosopumilus sp. NF5] | 1.00E-10 | 33 | Methyltransf_24 (3.82E-13; pfam13578; 35-137) |
| 41 | 270 | XP_001422290.1 | predicted protein [Ostreococcus lucimarinus CCE9901] | 2.00E-89 | 48 | SDR_e (2.20E-19; cd08946; 3-193): NADB_Rossmann superfamily (2.20E-19; cl21454; 3-193) |
| 42 | 256 | WP_028823613.1 | hypothetical protein [Proteobacteria bacterium JGI 0000113-P07] | 7.00E-28 | 34 | Glyco_transf_17 superfamily (1.68E-18; cl04706; 3-205) |
| 43 | 237 | ADQ91198.1 | hypothetical protein BpV2_031c [Bathycoccus sp. RCC1105 virus BpV2] | 3.00E-99 | 56 | Gly_transf_sug superfamily (3.40E-10; cl19952; 21-102) |
| 45 | 226 | AFC34938.1 | hypothetical protein OtV6_030 [Ostreococcus tauri virus RT-2011] | 1.00E-80 | 54 |  |
| **48** | **166** | **YP_001648292.1** | **hypothetical protein OsV5_216r [Ostreococcus virus OsV5]** | **4.00E-68** | **65** | **Pox_A22 superfamily (1.23E-04; cl04798; 1-150)** |
| **49** | **92** | **AET84535.1** | **hypothetical protein OLOG_00072 [Ostreococcus lucimarinus virus OlV4]** | **3.00E-06** | **43** |  |
| **50** | **70** | **YP_004061946.1** | **hypothetical protein MpV1_063c [Micromonas sp. RCC1109 virus MpV1]** | **1.00E-08** | **51** |  |
| 52 | 458 | YP_007674835.1 | hypothetical protein OLNG_00196 [Ostreococcus lucimarinus virus OlV5] | 0 | 67 | DEXDc (1.76E-12; cd00046; 106-229) |
| 53 | 375 | AET43741.1 | hypothetical protein MPWG_00257 [Micromonas pusilla virus PL1] | 3.00E-138 | 52 |  |
| **54** | **1394** | **YP_004061949.1** | **hypothetical protein MpV1_066 [Micromonas sp. RCC1109 virus MpV1]** | **0** | **34** |  |
| **55** | **64** | **YP_007676124.1** | **hypothetical protein MPVG_00056 [Micromonas pusilla virus 12T]** | **1.00E-06** | **41** |  |
| 56 | 379 | AET43745.1 | hypothetical protein MPWG_00261 [Micromonas pusilla virus PL1] | 7.00E-107 | 53 |  |
| **57** | **93** | **YP_004061704.1** | **hypothetical protein OlV1_071 [Ostreococcus lucimarinus virus 1]** | **3.00E-18** | **45** |  |
| **58** | **169** | **YP_007674831.1** | **hypothetical protein OLNG_00191 [Ostreococcus lucimarinus virus OlV5]** | **1.00E-79** | **69** |  |
| 59 | 346 | YP_004061953.1 | hypothetical protein MpV1_070 [Micromonas sp. RCC1109 virus MpV1] | 8.00E-124 | 49 | Capsid_NCLDV superfamily (1.72E-15; cl04526; 180-342) |
| 60 | 2068 | YP_004061707.1 | hypothetical protein OlV1_074 [Ostreococcus lucimarinus virus 1] | 2.00E-69 | 35 | V_Alix_like superfamily (7.01E-04; cl14654; 996-1200) |
| 61 | 1951 | YP_004061708.1 | hypothetical protein OlV1_075 [Ostreococcus lucimarinus virus 1] | 2.00E-172 | 35 | LbR-like superfamily (2.11E-07; cl17507; 477-616) |
| 62 | 414 | YP_004061659.1 | hypothetical protein OlV1_026c [Ostreococcus lucimarinus virus 1] | 8.00E-148 | 56 | Glycosyltransferase_GTB_type superfamily (1.15E-15; cl10013; 2-303) |
| **63** | **108** | **YP_003212990.1** | **hypothetical protein OTV1_167 [Ostreococcus tauri virus 1]** | **2.00E-13** | **33** |  |
| 64 | 1554 | YP_004061708.1 | hypothetical protein OlV1_075 [Ostreococcus lucimarinus virus 1] | 1.00E-48 | 31 | Peptidase_S74 (9.65E-05; pfam13884;1068-1120) |
| **66** | **81** | **AFC34985.1** | **hypothetical protein OtV6_077 [Ostreococcus tauri virus RT-2011]** | **8.00E-10** | **36** |  |
| **67** | **97** | **AFC34986.1** | **hypothetical protein OtV6_078 [Ostreococcus tauri virus RT-2011]** | **6.00E-10** | **48** |  |
| **68** | **180** | **AFC34987.1** | **hypothetical protein OtV6_079 [Ostreococcus tauri virus RT-2011]** | **2.00E-74** | **63** | **MutT (4.97E-05; COG0494; 1-60): Nudix_Hydrolase superfamily (4.97E-05; cl00447; 1-60)** |
| **69** | **77** | **YP_007676212.1** | **hypothetical protein MPVG_00147 [Micromonas pusilla virus 12T]** | **1.00E-05** | **41** |  |
| 70 | 480 | YP_004061966.1 | hypothetical protein MpV1_083 [Micromonas sp. RCC1109 virus MpV1] | 2.00E-164 | 49 |  |
| **71** | **132** | **YP_007676210.1** | **hypothetical protein MPVG_00145 [Micromonas pusilla virus 12T]** | **6.00E-16** | **41** |  |
| **72** | **130** | **AET84752.1** | **hypothetical protein OLOG_00301 [Ostreococcus lucimarinus virus OlV4]** | **4.00E-04** | **60** |  |
| **73** | **107** | **AET84753.1** | **hypothetical protein OLOG_00302 [Ostreococcus lucimarinus virus OlV4]** | **2.00E-14** | **45** |  |
| **74** | **91** | **YP_007676207.1** | **hypothetical protein MPVG_00142 [Micromonas pusilla virus 12T]** | **4.00E-09** | **43** |  |
| 75 | 226 | YP_004063508.1 | hypothetical protein OtV2_075 [Ostreococcus tauri virus 2] | 1.00E-81 | 61 | An_peroxidase (2.66E-03; pfam03098; 148-176) |
| 76 | 333 | YP_004061499.1 | hypothetical protein BpV1_069 [Bathycoccus sp. RCC1105 virus BpV1] | 9.00E-66 | 36 | NAT_SF (3.38E-03; cd04301; 77-140) |
| 77 | 223 | YP_004061971.1 | hypothetical protein MpV1_088 [Micromonas sp. RCC1109 virus MpV1] | 2.00E-90 | 61 | AdoMet_MTases (8.22E-16; cd02440; 26-125) |
| 78 | 140 | YP_004061972.1 | hypothetical protein MpV1_089 [Micromonas sp. RCC1109 virus MpV1] | 2.00E-56 | 61 | WLM superfamily (8.43E-06; cl07077; 63-121) |
| **79** | **188** | **YP_004061974.1** | **hypothetical protein MpV1_091c [Micromonas sp. RCC1109 virus MpV1]** | **5.00E-38** | **45** |  |
| 80 | 260 | AET84819.1 | hypothetical protein MPXG_00021 [Micromonas pusilla virus SP1] | 1.00E-136 | 69 | AAA_10 (6.54E-03; pfam12846; 83-173) |
| 81 | 418 | YP_004061976.1 | hypothetical protein MpV1_093 [Micromonas sp. RCC1109 virus MpV1] | 4.00E-141 | 51 | Capsid_NCLDV superfamily (1.30E-22; cl04526; 213-416) |
| 82 | 449 | YP_004061730.1 | hypothetical protein OlV1_097 [Ostreococcus lucimarinus virus 1] | 4.00E-131 | 47 | Capsid_NCLDV superfamily (1.26E-19; cl04526; 237-441) |
| **83** | **133** | **YP_004063520.1** | **hypothetical protein OtV2_087 [Ostreococcus tauri virus 2]** | **1.00E-35** | **48** |  |
| **85** | **93** | **AET84823.1** | **hypothetical protein MPXG_00025 [Micromonas pusilla virus SP1]** | **1.00E-27** | **57** |  |
| **86** | **331** | **AFK65909.1** | **hypothetical protein OLVG_00155 [Ostreococcus lucimarinus virus OlV6]** | **1.00E-67** | **40** |  |
| 87 | 373 | YP_004061735.1 | hypothetical protein OlV1_102c [Ostreococcus lucimarinus virus 1] | 1.00E-136 | 56 |  |
| **88** | **128** | **AET84826.1** | **hypothetical protein MPXG_00028 [Micromonas pusilla virus SP1]** | **2.00E-47** | **59** |  |
| 89 | 217 | YP_003212923.1 | hypothetical protein OTV1_100 [Ostreococcus tauri virus 1] | 4.00E-95 | 63 |  |
| **90** | **161** | **YP_004061984.1** | **hypothetical protein MpV1_101c [Micromonas sp. RCC1109 virus MpV1]** | **1.00E-55** | **56** |  |
| **91** | **72** | **YP_004061985.1** | **hypothetical protein MpV1_102c [Micromonas sp. RCC1109 virus MpV1]** | **6.00E-29** | **74** |  |
| 92 | 303 | AFC35013.1 | hypothetical protein OtV6_105c [Ostreococcus tauri virus RT-2011] | 5.00E-81 | 48 |  |
| 93 | 129 | YP_003212927.1 | hypothetical protein OTV1_104 [Ostreococcus tauri virus 1] | 9.00E-34 | 49 |  |
| **94** | **190** | **AFC35015.1** | **hypothetical protein OtV6_107c [Ostreococcus tauri virus RT-2011]** | **1.00E-32** | **39** |  |
| **95** | **135** | **AET84711.1** | **hypothetical protein OLOG_00256 [Ostreococcus lucimarinus virus OlV4]** | **1.00E-47** | **56** |  |
| 96 | 251 | AET84643.1 | hypothetical protein OLOG_00183 [Ostreococcus lucimarinus virus OlV4] | 3.00E-108 | 64 | PCNA (6.90E-43; cd00577; 6-248) |
| **97** | **200** | **YP_004061991.1** | **hypothetical protein MpV1_108c [Micromonas sp. RCC1109 virus MpV1]** | **1.00E-50** | **41** |  |
| 98 | 326 | YP_001648194.1 | hypothetical protein OsV5_117f [Ostreococcus virus OsV5] | 3.00E-128 | 59 | Pox_VLTF3 (4.59E-49; pfam04947; 149-325): Pox_VLTF3 superfamily (4.59E-49; cl04858; 149-325) |
| 100 | 235 | YP_001648091.1 | hypothetical protein OsV5_014f [Ostreococcus virus OsV5] | 2.00E-78 | 52 | AdoMet_MTases superfamily (1.01E-22; cl17173; 52-188) |
| 102 | 115 | YP_007676183.1 | hypothetical protein MPVG_00117 [Micromonas pusilla virus 12T] | 7.00E-46 | 67 | PDDEXK_3 (1.42E-31; pfam13366; 15-110) |
| 103 | 201 | YP_004063539.1 | prolyl 4-hydroxylase [Ostreococcus tauri virus 2] | 3.00E-72 | 59 | P4Hc (4.85E-33; smart00702; 33-193): 2OG-FeII_Oxy superfamily (4.85E-33; cl21496; 33-193) |
| 104 | 428 | AFC35026.1 | hypothetical protein OtV6_118c [Ostreococcus tauri virus RT-2011] | 5.00E-124 | 44 | Lebercilin (3.50E-03; pfam15619; 72-158) |
| **105** | **2218** | **YP_004061542.1** | **hypothetical protein BpV1_112c [Bathycoccus sp. RCC1105 virus BpV1]** | **3.00E-23** | **44** | **PTZ00121 (8.93E-08; PTZ00121; 282-980)** |
| 106 | 253 | YP_003212939.1 | hypothetical protein OTV1_116 [Ostreococcus tauri virus 1] | 5.00E-101 | 58 | Patatin (5.17E-27; pfam01734; 4-172) |
| 107 | 125 | AFC35030.1 | hypothetical protein OtV6_122c [Ostreococcus tauri virus RT-2011] | 5.00E-45 | 58 | DUF3339 superfamily (6.30E-03; cl13288; 9-64) |
| **108** | **122** | **AFC35031.1** | **hypothetical protein OtV6_123c [Ostreococcus tauri virus RT-2011]** | **8.00E-12** | **35** |  |
| **109** | **122** | **YP_003212942.1** | **hypothetical protein OTV1_119 [Ostreococcus tauri virus 1]** | **6.00E-39** | **59** |  |
| **110** | **148** | **AET84627.1** | **hypothetical protein OLOG_00167 [Ostreococcus lucimarinus virus OlV4]** | **4.00E-43** | **54** |  |
| **111** | **195** | **AET84626.1** | **hypothetical protein OLOG_00166 [Ostreococcus lucimarinus virus OlV4]** | **1.00E-43** | **47** |  |
| **112** | **469** | **AET84625.1** | **hypothetical protein OLOG_00165 [Ostreococcus lucimarinus virus OlV4]** | **2.00E-172** | **52** |  |
| 113 | 140 | YP_001648221.1 | hypothetical protein OsV5_144f [Ostreococcus virus OsV5] | 1.00E-43 | 56 | SWIB superfamily (1.94E-12; cl02489; 51-133) |
| 114 | 228 | YP_004061782.1 | hypothetical protein OlV1_149c [Ostreococcus lucimarinus virus 1] | 4.00E-92 | 63 | RIBOc (3.70E-46; cd00593; 24-147) |
| 115 | 237 | YP_007676170.1 | hypothetical protein MPVG_00104 [Micromonas pusilla virus 12T] | 4.00E-114 | 70 | YqaJ (1.48E-39; pfam09588; 20-145): YqaJ superfamily (1.48E-39; cl09232; 20-145) |
| **118** | **202** | **AFC35042.1** | **hypothetical protein OtV6_134c [Ostreococcus tauri virus RT-2011]** | **9.00E-67** | **52** |  |
| 119 | 1166 | AET84860.1 | ribonucleotide reductase [Micromonas pusilla virus SP1] | 0 | 70 | RNR_I (0; cd01679; 619-1143) |
| 121 | 131 | YP_004062024.1 | hypothetical protein MpV1_141 [Micromonas sp. RCC1109 virus MpV1] | 7.00E-50 | 59 |  |
| 122 | 103 | YP_004061773.1 | hypothetical protein OlV1_140c [Ostreococcus lucimarinus virus 1] | 2.00E-44 | 71 | NTP-PPase superfamily (4.52E-10; cl16941; 10-84) |
| **123** | **151** | **YP_001648234.1** | **hypothetical protein OsV5_157f [Ostreococcus virus OsV5]** | **1.00E-08** | **30** |  |
| **124** | **162** | **AET43598.1** | **hypothetical protein MPWG_00109 [Micromonas pusilla virus PL1]** | **2.00E-41** | **46** |  |
| 125 | 291 | AFC35049.1 | hypothetical protein OtV6_141 [Ostreococcus tauri virus RT-2011] | 3.00E-83 | 46 | TBP_TLF superfamily (3.85E-08; cl08263; 100-235) |
| **126** | **79** | **YP_004062029.1** | **hypothetical protein MpV1_146 [Micromonas sp. RCC1109 virus MpV1]** | **4.00E-29** | **65** |  |
| 127 | 117 | YP_001648241.1 | hypothetical protein OsV5_164f [Ostreococcus virus OsV5] | 4.00E-38 | 50 | RING (3.90E-11; cd00162; 2-48) |
| 128 | 237 | AET84869.1 | hypothetical protein MPXG_00071 [Micromonas pusilla virus SP1] | 2.00E-84 | 58 | LysM (5.17E-03; pfam01476; 126-166) |
| **129** | **70** | **AET43593.1** | **hypothetical protein MPWG_00104 [Micromonas pusilla virus PL1]** | **1.00E-17** | **50** |  |
| **130** | **100** | **YP_001648244.1** | **hypothetical protein OsV5_167r [Ostreococcus virus OsV5]** | **2.00E-28** | **56** | **Thioredoxin_like superfamily (1.30E-03; cl00388; 43-95)** |
| 131 | 236 | AFC35059.1 | hypothetical protein OtV6_151c [Ostreococcus tauri virus RT-2011] | 5.00E-94 | 62 |  |
| 133 | 249 | YP_003212996.1 | hypothetical protein OTV1_173 [Ostreococcus tauri virus 1] | 6.00E-110 | 64 | DNA_BRE_C superfamily (2.80E-22; cl00213; 50-247) |
| 135 | 390 | YP_004062126.1 | hypothetical protein MpV1_243 [Micromonas sp. RCC1109 virus MpV1] | 4.00E-44 | 37 | Laminin_G_3 (2.90E-03; pfam13385; 120-270) |
| **137** | **203** | **YP_004061760.1** | **hypothetical protein OlV1_127c [Ostreococcus lucimarinus virus 1]** | **5.00E-32** | **54** | **TIM_phosphate_binding superfamily (1.19E-03; cl21457; 87-131)** |
| 138 | 176 | YP_007674742.1 | hypothetical protein OLNG_00100 [Ostreococcus lucimarinus virus OlV5] | 8.00E-49 | 51 |  |
| 139 | 417 | YP_007676142.1 | ribonucleoside-diphosphate reductase small subunit [Micromonas pusilla virus 12T] | 0 | 68 | RNRR2 (3.48E-113; cd01049; 112-386) |
| 140 | 308 | AFK66122.1 | hypothetical protein OMVG_00122 [Ostreococcus lucimarinus virus OlV3] | 3.00E-22 | 34 | FA58C superfamily (7.62E-05; cl19067; 105-181) |
| 141 | 226 | AFK66093.1 | hypothetical protein OMVG_00093 [Ostreococcus lucimarinus virus OlV3] | 5.00E-67 | 49 | DUF925 superfamily (6.79E-03; cl01435; 5-68) |
| 142 | 234 | YP_008052449 | ribonuclease H [Phaeocystis globosa virus] | 3.06E-42 | 48 |  |
| 143 | 480 | YP_004062126.1 | hypothetical protein MpV1_243 [Micromonas sp. RCC1109 virus MpV1] | 1.00E-26 | 40 |  |
| 144 | 516 | YP_004062126.1 | hypothetical protein MpV1_243 [Micromonas sp. RCC1109 virus MpV1] | 2.00E-21 | 37 | FliL superfamily (3.61E-04; cl00681; 1-53) |
| **145** | **531** | **YP_004062126.1** | **hypothetical protein MpV1_243 [Micromonas sp. RCC1109 virus MpV1]** | **5.00E-18** | **34** |  |
| 147 | 429 | YP_001648266.1 | hypothetical protein OsV5_190f [Ostreococcus virus OsV5] | 0 | 70 | Capsid_NCLDV (2.53E-64; pfam04451; 190-425) |
| 149 | 272 | WP_048189474.1 | hypothetical protein [Thaumarchaeota archaeon SAT1] | 2.00E-59 | 41 | Dam (5.56E-34; COG0338; 7-262) |
| 150 | 222 | YP_009174116.1 | Hypothetical protein [Yellowstone lake mimivirus] | 7.00E-18 | 37 |  |
| 152 | 652 | YP_001648264.1 | hypothetical protein OsV5_188r [Ostreococcus virus OsV5] | 0 | 48 | primase_Cterm superfamily (8.68E-15; cl20060; 380-538) |
| **154** | **134** | **YP_004061794.1** | **hypothetical protein OlV1_162c [Ostreococcus lucimarinus virus 1]** | **6.00E-11** | **35** |  |
| **155** | **99** | **YP_004063581.1** | **hypothetical protein OtV2_148 [Ostreococcus tauri virus 2]** | **1.00E-09** | **31** |  |
| 156 | 253 | WP_036271039.1 | MULTISPECIES: hypothetical protein, partial [Methylobacterium] | 2.00E-27 | 40 | Methyltransf_24 (5.13E-07; pfam13578; 127-216) |
| 157 | 197 | AFC35071.1 | hypothetical protein OtV6_163 [Ostreococcus tauri virus RT-2011] | 2.00E-69 | 53 | CYTH-like_Pase superfamily (1.67E-15; cl11964; 25-196) |
| 158 | 255 | YP_004062044.1 | hypothetical protein MpV1_161c [Micromonas sp. RCC1109 virus MpV1] | 2.00E-91 | 50 | Peptidase_C19 (3.03E-16; cd02257; 4-234) |
| 159 | 303 | YP_004063578.1 | hypothetical protein OtV2_145 [Ostreococcus tauri virus 2] | 1.00E-87 | 47 | Adenylation_DNA_ligase_like superfamily (8.78E-33; cl12015; 22-201): |
| **160** | **217** | **YP_004062042.1** | **hypothetical protein MpV1_159 [Micromonas sp. RCC1109 virus MpV1]** | **3.00E-62** | **48** |  |
| 161 | 249 | YP_004062041.1 | hypothetical protein MpV1_158 [Micromonas sp. RCC1109 virus MpV1] | 1.00E-122 | 67 |  |
| 162 | 329 | YP_004061757.1 | hypothetical protein OlV1_124c [Ostreococcus lucimarinus virus 1] | 2.00E-135 | 59 | CYCLIN (2.30E-03; cd00043; 136-220) |
| 163 | 183 | KHG00790.1 | Transcription factor MYB1R1 [Gossypium arboreum] | 6.00E-20 | 53 | SANT (9.40E-11; cd00167; 25-69) |
| 164 | 99 | YP_004061942.1 | hypothetical protein MpV1_059 [Micromonas sp. RCC1109 virus MpV1] | 1.00E-30 | 60 |  |
| **165** | **99** | **YP_007676132.1** | **hypothetical protein MPVG_00064 [Micromonas pusilla virus 12T]** | **1.00E-25** | **55** |  |
| 166 | 256 | WP_028823908.1 | hypothetical protein [Proteobacteria bacterium JGI 0000113-P07] | 8.00E-18 | 28 | Glyco_transf_25 (3.63E-18; cd06532; 4-162) |
| **167** | **234** | **AFK66235.1** | **hypothetical protein OMVG_00241 [Ostreococcus lucimarinus virus OlV3]** | **1.00E-11** | **30** |  |
| 168 | 402 | WP_012312618.1 | hypothetical protein [Pseudomonas putida] | 3.00E-04 | 29 |  |
| **169** | **270** | **YP_003212998.1** | **hypothetical protein OTV1_175 [Ostreococcus tauri virus 1]** | **9.00E-91** | **52** | **Glyco_transf_25 superfamily (1.77E-11; cl01298; 30-134)** |
| 170 | 525 | WP_005195299.1 | hypothetical protein [Acinetobacter sp. NIPH 298] | 2.00E-17 | 29 | Gly_transf_sug superfamily (4.10E-06; cl19952; 58-140) |
| **171** | **226** | **YP_003212994.1** | **hypothetical protein OTV1_171 [Ostreococcus tauri virus 1]** | **5.00E-81** | **61** |  |
| 172 | 116 | AFC34969.1 | hypothetical protein OtV6_061 [Ostreococcus tauri virus RT-2011] | 1.00E-18 | 47 | Ion_trans_2 (1.52E-06; pfam07885; 13-84) |
| **174** | **88** | **YP_001648096.1** | **hypothetical protein OsV5_019r [Ostreococcus virus OsV5]** | **2.00E-35** | **71** |  |
| 175 | 120 | XP_009314682.1 | E3 ubiquitin-protein ligase RNF5 [Trypanosoma grayi] | 2.00E-05 | 41 | RING (3.37E-05; cd00162; 32-69) |
| **176** | **132** | **AET84999.1** | **hypothetical protein MPXG_00201 [Micromonas pusilla virus SP1]** | **2.00E-37** | **52** | **HAD_like superfamily (2.69E-04; cl21460; 21-118)** |
| 178 | 167 | AET85000.1 | transcription elongation factor [Micromonas pusilla virus SP1] | 6.00E-52 | 49 | Zn-ribbon_TFIIS (3.45E-18; cd13749; 122-167) |
| **179** | **73** | **AET43709.1** | **hypothetical protein MPWG_00223 [Micromonas pusilla virus PL1]** | **2.00E-12** | **49** | **Rifin_STEVOR superfamily (1.52E-03; cl14106; 5-22)** |
| **180** | **81** | **YP_004061666.1** | **hypothetical protein OlV1_033c [Ostreococcus lucimarinus virus 1]** | **5.00E-19** | **49** |  |
| 181 | 517 | YP_004061667.1 | hypothetical protein OlV1_034c [Ostreococcus lucimarinus virus 1] | 0 | 65 | Asn_Synthase_B_C (3.04E-55; cd01991; 203-429) |
| **182** | **213** | **YP_004063456.1** | **hypothetical protein OtV2_023 [Ostreococcus tauri virus 2]** | **2.00E-21** | **34** |  |
| 183 | 209 | AFC34937.1 | hypothetical protein OtV6_029 [Ostreococcus tauri virus RT-2011] | 3.00E-85 | 59 | PhoH (3.58E-69; pfam02562; 10-208) |
| 185 | 122 | YP_003213016.1 | hypothetical protein OTV1_193 [Ostreococcus tauri virus 1] | 2.00E-37 | 55 |  |
| 188 | 373 | YP_004062102.1 | hypothetical protein MpV1_219c [Micromonas sp. RCC1109 virus MpV1] | 9.00E-145 | 54 | Capsid_NCLDV superfamily (9.33E-30; cl04526; 196-369) |
| 189 | 919 | YP_004061851.1 | hypothetical protein OlV1_219 [Ostreococcus lucimarinus virus 1] | 0 | 60 | POLBc superfamily (9.79E-120; cl10023; 453-865) |
| **190** | **127** | **AET84948.1** | **hypothetical protein MPXG_00150 [Micromonas pusilla virus SP1]** | **4.00E-15** | **39** |  |
| **191** | **104** | **YP_004061868.1** | **hypothetical protein OlV1_236 [Ostreococcus lucimarinus virus 1]** | **2.00E-32** | **56** |  |
| 193 | 131 | YP_004062105.1 | hypothetical protein MpV1_222 [Micromonas sp. RCC1109 virus MpV1] | 5.00E-53 | 62 |  |
| 194 | 489 | YP_007674676.1 | hypothetical protein OLNG_00030 [Ostreococcus lucimarinus virus OlV5] | 3.00E-173 | 53 | Capsid_NCLDV superfamily (1.17E-07; cl04526; 237-416) |
| 195 | 1070 | AFC35140.1 | hypothetical protein OtV6_232c [Ostreococcus tauri virus RT-2011] | 0 | 59 | HATPase_c (1.03E-04; cd00075; 54-165) |
| **197** | **121** | **YP_004061857.1** | **hypothetical protein OlV1_225 [Ostreococcus lucimarinus virus 1]** | **1.00E-39** | **63** | P-loop_NTPase superfamily (1.33E-03; cl21455; 19-65) |
| **199** | **74** | **AET84957.1** | **hypothetical protein MPXG_00159 [Micromonas pusilla virus SP1]** | **3.00E-23** | **65** |  |
| 200 | 320 | YP_007676308.1 | hypothetical protein MPVG_00244 [Micromonas pusilla virus 12T] | 3.00E-133 | 61 | Glycosyltransferase_GTB_type (1.56E-07; cd01635; 138-236) |
| 201 | 103 | YP_004061861.1 | hypothetical protein OlV1_229 [Ostreococcus lucimarinus virus 1] | 1.00E-15 | 55 |  |
| **202** | **121** | **YP_004063625.1** | **predicted host protein [Ostreococcus tauri virus 2]** | **9.00E-24** | **45** |  |
| **203** | **243** | **AET43506.1** | **hypothetical protein MPWG_00016 [Micromonas pusilla virus PL1]** | **4.00E-79** | **53** |  |
| **207** | **84** | **AFK66008.1** | **hypothetical protein OMVG_00002 [Ostreococcus lucimarinus virus OlV3]** | **4.00E-13** | **46** |  |
| **209** | **420** | **YP_003212829.1** | **hypothetical protein OTV1_005 [Ostreococcus tauri virus 1]** | **3.00E-88** | **39** | **DEDDh (2.18E-30; cd06127; 4-180)** |
| **214** | **155** | **AET84713.1** | **hypothetical protein OLOG_00258 [Ostreococcus lucimarinus virus OlV4]** | **3.00E-18** | **47** |  |
| 217 | 116 | YP_004062100.1 | hypothetical protein MpV1_217c [Micromonas sp. RCC1109 virus MpV1] | 4.00E-38 | 60 | RHOD (4.83E-25; cd00158; 30-113) |
| 219 | 330 | WP_036373161.1 | hypothetical protein, partial [Mycobacterium austroafricanum] | 9.00E-23 | 30 | HA (9.48E-10; pfam03457; 187-248) |

Light blue for NCLDVs hits (n=155), light green for eukaryote hit (n=4), red for bacteria hit (n=6), and yellow for archaea hit (n=2); **Light blue in bold, ORFs with virus-hits only**.

**Table S4** GenBank accession number of all the sequences used in the phylogeny

| **Sequence name in Figure 2B** | **GenBank accession #s** |
| --- | --- |
|  |  |
| Ostreococcus mediterraneus virus 1 | YP_009172985.1 |
| Ostreococcus tauri virus 5 | YP_001648316.1 |
| Ostreococcus tauri virus 1 | YP_003213031.1 |
| Ostreococcus lucimarinus virus 2 | YP_009172731.1 |
| Ostreococcus tauri virus 2 | YP_004063640.1 |
| Ostreococcus lucimarinus virus 1 | YP_004061851.1 |
| Ostreococcus lucimarinus virus 7 | YP_009173227.1 |
| Ostreococcus tauri virus RT-2011 | AFC35136.1 |
| Micromonas sp. RCC1109 virus MpV1 | YP_004062103.1 |
| Micromonas pusilla virus SP1 | AET84947.1 |
| Micromonas pusilla virus PL1 | AET43521.1 |
| Micromonas pusilla virus 12T | YP_007676285.1 |
| Bathycoccus sp. RCC1105 virus BpV1 | YP_004061614.1 |
| Bathycoccus sp. RCC1105 virus BpV2 | ADQ91356.1 |
| DSLPV1 | KY747489 |
| Yellowstone Lake phycodnavirus 1 | YP_009174732.1 |
| Yellowstone Lake phycodnavirus 2 | YP_009174598.1 |
| Acanthocystis turfacea Chlorella virus 1 | YP_001427279.1 |
| Paramecium bursaria Chlorella virus CVA-1 | AGE50367.1 |
| Paramecium bursaria Chlorella virus 1 | NP_048532.2 |
| Paramecium bursaria Chlorella virus NY2A | YP_001497445.1 |
| Paramecium bursaria Chlorella virus NYs1 | AGE58659.1 |
| Phaeocystis globosa virus 16T | YP_008052566.1 |
| **Sequence name in Figure 3C** | |
|  |  |
| Ectocarpus siliculosus | CBN79887.1 |
| Noccaea caerulescens | JAU99846.1 |
| Phaeodactylum tricornutum CCAP 1055/1 | XP_002181134.1 |
| Fragilariopsis cylindrus CCMP1102 | OEU09978.1 |
| Thalassiosira pseudonana CCMP1335 | XP_002293701.1 |
| Ostreococcus lucimarinus CCE9901 | XP_001419259.1 |
| Micromonas commode | XP_002502604.1 |
| Ostreococcus tauri | XP_003082036.1 |
| Klebsormidium flaccidum | GAQ89306.1 |
| Micromonas pusilla CCMP1545 | XP_003057560.1 |
| Guillardia theta CCMP2712 | XP_005830219.1 |
| Bathycoccus prasinos | XP_007513760.1 |
| Chondrus crispus | XP_005711872.1 |
| Fucus serratus | CAB82768.1 |
| Coccomyxa subellipsoidea C-169 | XP_005643646.1 |
| Chlorella variabilis | XP_005843780.1 |
| Polyblepharides amylifera | BAS30515.1 |
| Galdieria sulphuraria | XP_005702721.1 |
| Monoraphidium neglectum | XP_013898030.1 |
| Brazilian marseillevirus | YP_009238943.1 |
| Lausannevirus | YP_004347349.1 |
| Tunisvirus fontaine2 | AHC55030.1 |
| Tokyovirus A1 | YP_009254842.1 |
| Cannes 8 virus | AGV01787.1 |
| Marseillevirus marseillevirus | YP_003407137.1 |
| **Sequence name in Figure S2** | |
|  |  |
| Prochlorococcus phage P-SSM2 | YP_214490.1 |
| Synechococcus phage S-SSM7 | YP_004324307.1 |
| Candidatus Pelagibacter sp. IMCC9063 | WP_013695256.1 |
| Candidatus Yanofskybacteria bacterium | OGN40942.1 |
| Ostreococcus lucimarinus CCE9901 | XP_001422290.1 |
| Parcubacteria group bacterium | KKW46709.1 |
| Smithella sp. SDB | KQC10121.1 |
| Guillardia theta CCMP2712 | XP_005820425.1 |
| Tritrichomonas foetus | OHS95297.1 |
| Tritrichomonas foetus 2 | OHT10879.1 |


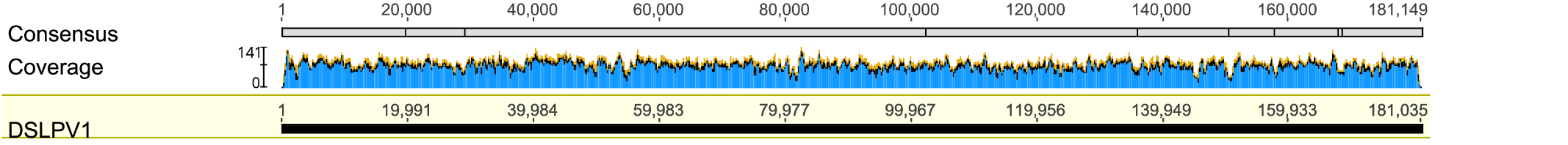


**Figure S1 Coverage of sequence assembly of DSLPV1.** The blue area represents abundance of reads mapped to the genome. The number of the maximum and minimum of mapped reads are showed in the left scale bar.


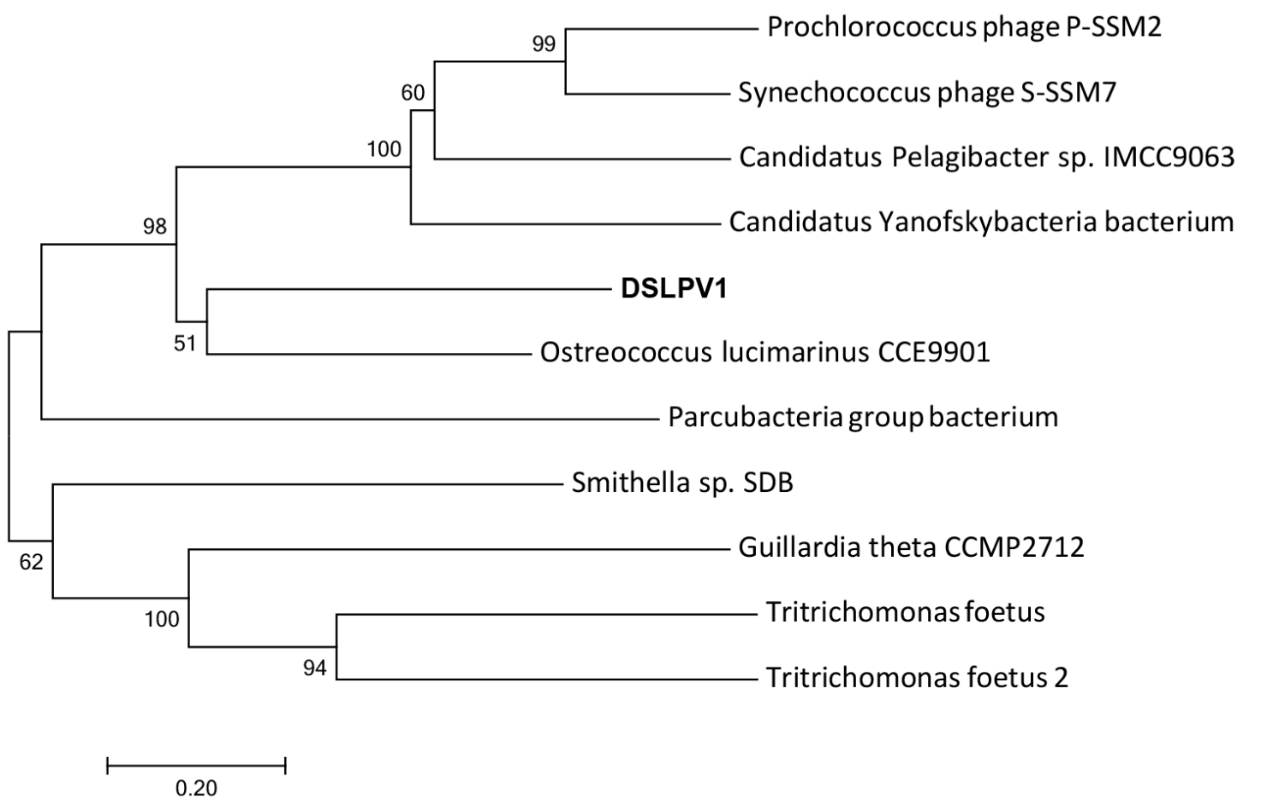


**Figure S2. Maximum likelihood phylogenetic tree of** **the NAD-dependent epimerase/dehydratase proteins.** The scale bar indicates a distance of 0.2 fixed mutations per amino acid position. GenBank accession numbers of the NAD-dependent epimerase/dehydratase sequences used for this tree are listed in Table S4. Only more than 50% of bootstrap value is shown in the tree.
